# Exploring the Impact of Student-Staff Partnerships in Higher Education: A Realist Review Protocol.

**DOI:** 10.12688/f1000research.163068.1

**Published:** 2025-04-07

**Authors:** Seán Paul Teeling, Naomi McAreavey, Rachel Farrell, Olive Lennon

**Affiliations:** 1 University College Dublin School of Nursing Midwifery and Health Systems, Dublin, Leinster, Ireland; 2 University College Dublin School of English Drama and Film, Dublin, Leinster, Ireland; 3 University College Dublin School of Education, Dublin, Leinster, Ireland; 4 University College Dublin School of Public Health Physiotherapy and Sports Science, Dublin, Leinster, Ireland

**Keywords:** Student-staff partnership, higher education, realist review, programme theory, engagement strategies, context-mechanism-outcome, institutional change, policy development

## Abstract

**Background:**

Student-staff partnership (SSP) in higher education (HE) is rooted in democratic education and critical pedagogy, advocating for active student engagement. While widely recognised for fostering student success, SSP initiatives vary in definition, implementation, and impact. In Ireland, national policy has positioned SSP as central to student engagement, yet there is limited synthesis of the contextual factors and mechanisms influencing its success. Existing literature highlights benefits such as increased engagement and enhanced learning but often overlooks challenges, including power dynamics, resistance to change, and inconsistent institutional commitment.

**Aim:**

This protocol outlines the methodology for a realist review, forming the first stage of a broader realist inquiry into SSP initiatives. The review will synthesise existing evidence to understand how, why, and in what contexts SSP initiatives succeed or face challenges in HE. Specifically, it will identify the contextual factors, mindsets, and engagement strategies that enable meaningful partnerships, as well as mechanisms for overcoming barriers. The review will generate initial programme theories (IPTs) to inform a subsequent realist evaluation within University College Dublin (UCD).

**Method:**

A realist review methodology will be used to explore the interactions between context, mechanisms, and outcomes in SSP initiatives. The study will follow a five-stage realist review process: scoping the literature, developing IPTs, systematically reviewing evidence, synthesising findings, and refining theories with expert input. The Context-Mechanism-Outcome Configuration (CMOC) framework will guide the analysis.

**Conclusion:**

This protocol sets out the approach for developing evidence-informed programme theories on SSP. These theories will underpin a subsequent realist evaluation within UCD to refine SSP implementation strategies. Findings will inform institutional strategies, policy development, and academic practice, with dissemination through academic and practitioner-focused outputs.

## Background

The concept of student-staff partnership (SSP) is rooted in the advocacy for more democratic approaches to education and critical pedagogy ( Bovill, 2012). This has significantly influenced the current understanding of SSP ( Bovill, 2019, p. 6). Although SSP is also known as Students as Partners (SaP), the authors use SSP for this study because it recognises both parties in the partnership ( Cook-Sather et al., 2018). The emergence of SSP marked a departure from consumeristic and neo-liberal educational paradigms, prioritising democratic values and social justice ( Lubicz-Nawrocka and Bao, 2025).

In Ireland, the origins of SSP can be traced back to the 2016 report by the Higher Education Authority (HEA) titled “Enhancing Student Engagement in Decision-Making.” This report promoted students as partners in Irish Higher Education (HE) rather than treating them as consumers or passive recipients of their education. It outlined principles for enhancing student-staff engagement and fostering partnership in HE decision-making. Subsequently, the National Student Engagement Programme has been instrumental in translating this vision into action nationally ( Higher Education Authority, 2021). “Student success” was one of four strategic priorities of the ( National Forum for the Enhancement of Teaching and Learning in Higher Education 2019), the national body responsible for leading and advising on the enhancement of teaching and learning in Irish Higher Education (HE) (2019-21), with its vision of success developed in partnership with students ( National Forum, 2019). The National Forum came under the aegis of the HEA in January 2022, and the HEA’s Student Engagement and Teaching & Learning Committee was established after that. SSP remains at the heart of national HE policy in Ireland.

Many universities in Ireland and globally have embraced SSP programmes, values, and objectives to varying degrees. Furthermore, the literature suggests a general consensus among scholars and educators regarding the numerous benefits of partnership; however, despite this consensus, there remains variability in how central values and concepts are defined across different publications ( Mercer-Mapstone et al., 2017).

One widely accepted definition of partnership, articulated by Bovill, Cook-Sather, and Felten (2017), describes it as a “collaborative, reciprocal process through which all participants have the opportunity to contribute equally, although not necessarily in the same ways, to curricular or pedagogical conceptualisation, decision making, implementation, investigation, or analysis” (pp 6-7). However, this definition may overlook non-academic forms of partnership, including those between students and non-academic staff members, or in areas such as civic engagement, research, and enhancing student experiences. Within partnership literature, non-academic partnerships are notably underrepresented ( Healey, Flint, and Harrington, 2014; Mercer-Mapstone et al., 2017). To avoid overly prescriptive definitions regarding the outcomes or focuses of partnership practices, Healey, Flint, and Harrington (2014) advocate for an open-ended understanding of partnership as a "process" of student engagement in HE rather than merely an outcome or product (p. 14). Furthermore, while their model primarily addresses partnership processes in teaching and learning settings, it remains compatible with and not restrictive of non-academic partnership practices.

The limited reporting on factors that contribute to the success of SSP schemes and the challenges they encounter presents a significant gap in HE research. Key elements such as institutional culture, the readiness of faculty and students, and the adequacy of support structures are often inadequately documented. Positive outcomes, like increased student engagement and enhanced learning experiences, are frequently highlighted, while challenges, including power imbalances, resistance to change, and logistical hurdles, receive less attention ( Bovill et al., 2017; Carroll, 2023). More recent scholarship has argued for the importance of mindset ( Gauthier, 2020; Rideau, 2022), cultural context ( Cook-Sather et al., 2020); representation ( Casey, 2024); equity ( Bindra et al., 2018; De Bie et al., 2021); managing disagreements ( Abbot & Cook, 2020) and fostering successful and sustainable SSP. However, this emerging research has not yet been fully synthesised.

Healey, Flint, and Harrington (2014) emphasise that successful SSP require mutual respect and a genuine commitment from both students and staff. Yet, the literature does not always fully explore these nuanced relational dynamics. Similarly, Bovill et al. (2017) point out that the complexities of implementing such partnerships, such as aligning them with institutional policies and managing expectations, are often underreported. Addressing these gaps is crucial for developing more effective and sustainable SSP in tertiary education. There is no clear understanding of the contexts in which SSP interventions work most effectively or of the mechanisms that encourage student, staff and faculty engagement in those interventions that lead to specific anticipated outcomes. That is the purpose of our research.

## Study objectives and location

This study is being undertaken by a team of four fellows in teaching and academic development at University College Dublin (UCD). UCD is Ireland’s largest university and a leading global institution. It hosts over 38,000 students from 152 countries and is ranked among the top 1% of HEIs worldwide. It offers diverse disciplines across six colleges, including Arts and Humanities, Social Sciences and Law, and Health and Agricultural Sciences. The fellows come from three of the six colleges, representing the disciplines of Education, English, Nursing, and Physiotherapy. Student partnership is central to UCD’s strategy to 2030, and the institution set the theme for our fellowship project, which it sponsors ( University College Dublin, 2024).

Our research is driven by the need to develop a well-defined, inclusive, and context-sensitive model for SSP in UCD and beyond. It seeks to understand how, within the context of UCD, the university can enable students, professional staff, and faculty to engage in meaningful SSP activities to improve the university experience for all. Our specific research questions are:

1. What contextual factors (including institutional culture and structures) enable students, professional staff and faculty to engage in meaningful SSP initiatives to improve the university experience for all?
2. What mindsets and modes of engagement (attitudes and ways of interacting) enable students, professional staff and faculty to engage in meaningful SSP initiatives to improve the university experience for all?
3. What enables good management of the challenges that arise in meaningful SSP initiatives?

Our project aims to foster effective and sustainable SSP initiatives that can be replicated across various educational contexts by addressing underreported factors, enhancing understanding of relational dynamics, and providing practical outputs.

This protocol paper outlines the rationale and methods for using realist review to explore the complex dynamics of SSP initiatives in HE.

## Methods

### Realist inquiry

We have adopted realist inquiry as the method for this study because it effectively unpacks the mechanisms of complex systems, providing actionable insights that benefit all stakeholders ( Wong et al., 2013). Realist inquiry is a methodology designed to understand complex interventions by exploring the “how” and “why” behind outcomes in specific contexts ( Wong et al., 2017). Unlike traditional evaluation approaches that focus on whether interventions work, realist inquiry delves deeper into the mechanisms that produce outcomes, considering the influence of various contexts ( Teeling et al., 2021). It is beneficial for evaluating interventions in dynamic, multifaceted settings, such as healthcare, where multiple factors influence results ( Pawson, 2013a; Wong et al., 2013; Manzano et al., 2017).

A fundamental principle of realist inquiry is that interventions do not work in a vacuum. Their success or failure is not merely a result of the intervention itself. However, it is significantly influenced by the context in which they are implemented and the mechanisms activated within that context ( Wong et al., 2017). This approach emphasises understanding *why* and *how* interventions work, offering a more nuanced understanding than simple cause-effect evaluations ( Pawson & Tilley, 1997).

Realist inquiry consists of two main components: realist review and realist evaluation, each serving distinct but complementary roles ( Teeling et al., 2021). Realist reviews are used to synthesise existing evidence and develop programme theories, while realist evaluations test and refine these theories in specific settings ( Pawson et al., 2005; Marchal, 2012). Together, they offer a comprehensive framework for understanding interventions' complexities and outcomes. This protocol paper outlines the rationale and methods for using realist review to explore the complex dynamics of SSP in HE.

### Realist review

Realist review, or realist synthesis, is a methodological approach to review existing evidence and refine theories about how and why interventions work ( Pawson et al., 2005). This approach is especially suited for synthesising evidence from diverse studies and understanding the complexities of interventions in real-world settings. Realist review is theory-driven and aims to generate insights that can inform practice and policy by identifying the mechanisms through which interventions achieve (or fail to achieve) their outcomes ( Wong et al., 2013).

Realist reviews are guided by the principle that interventions do not work in a vacuum; their success or failure depends on the context in which they are implemented and the mechanisms activated in that context. The review focuses on understanding the interactions between the context, mechanisms, and outcomes (CMO), thus offering a more nuanced understanding of how SSPs lead to different outcomes in different settings ( Wong et al., 2013).

One of the core concepts of realist inquiry is the CMOC (Context-Mechanism-Outcome Configuration) framework, which describes and explains how an intervention produces its effects. The CMOC approach is based on the idea that an intervention's success or failure depends not only on the intervention itself but also on how it interacts with its context and activates specific mechanisms to produce outcomes ( Pawson, 2013b; Wong et al., 2013).

• Context (C) refers to the conditions, settings, or environments in which an intervention occurs. This includes factors like organisational structures, cultural settings, policies, and stakeholder relationships. The context can either facilitate or hinder the mechanisms that produce outcomes. In realist evaluation, understanding context is crucial because the same intervention can have different effects depending on where and how it is implemented ( Pawson, 2013a; Manzano, 2016).
• Mechanism (M): Mechanisms are the processes or underlying drivers that explain how an intervention produces outcomes. Mechanisms are not always directly observable but can be inferred from how context interacts with the intervention. They reflect the responses of individuals or groups to an intervention. For example, a mechanism might be the activation of a sense of ownership or responsibility among staff members when they are involved in decision-making, which then influences the effectiveness of the intervention ( Pawson & Tilley, 1997; Wong et al., 2013).
• Outcome (O): Outcomes refer to the consequences or effects of an intervention. They can be intended or unintended and vary depending on the context and the mechanisms activated. In realist inquiry, the focus is on whether an outcome is achieved and on understanding *why* and *how* it was achieved (or not) in a particular context ( Pawson, 2013a, 2013b; Greenhalgh et al., 2017).

Combining these three elements—context, mechanism, and outcome—forms the CMOC configuration, which describes how an intervention produces results in specific settings. This configuration provides a deeper understanding of the intervention by explaining the underlying causal processes ( Pawson, 2013a). Understanding CMOCs is essential because they allow for predictions about the conditions under which interventions may or may not work and for whom ( Pawson, 2013a).

A realist review also serves as a foundational step in realist evaluation by developing programme theories. These theories hypothesise about the mechanisms at play and the contexts in which they are activated. They are then tested and refined in real world situations through further research, such as a realist evaluation ( Pawson, 2013a). The realist review described in this protocol paper will inform a subsequent realist evaluation.

### The use of realist review in higher education

Realist review is emerging as a valuable approach in HE for evaluating complex initiatives that aim to foster improvement and well-being within institutions.

Educational theories that explain the application, interpretation, and purpose of learning and education underpin usual teaching and learning activities ( Artino and Konopasky (2018). These theoretical concepts help explain the learning process, inform educational approaches, curricula, and assessments ( Khalil & Elkhider, 2016) and enable evaluation of methods of teaching ( Albert et al., 2007; Rees & Monrouxe, 2010). While systematic reviews have examined the concept of SSP in teaching and learning ( Mercer et al., 2017) and developed theories related to the partnership praxis ( Matthews et al., 2018), a broader study design is required to explain how and why SSP might work and in what contexts they are successful ( Suri & Clarke, 2009; Wong et al., 2012). Realist review is particularly suited to this purpose and is an emerging area in the scholarship of teaching and learning that seeks to understand and explain to what extent, how, why, for whom, and in what circumstances complex educational interventions produce their effects. As a methodology, it can provide an important step for educators to understand how to tailor educational offerings and the university experience to meet the needs of different learner groups and academic faculty and staff. In addition, developing programme theory in teaching and learning facilitates a localised realist evaluation specific to local contexts.

Realist review in education has previously been used to help develop programme theory related to the factors affecting teaching and learning for medicines supply management training ( Brown et al., 2013), to study how structural and cultural elements combine to build a “quality culture” in HE ( Bendermacher et al., 2017) and as a means to examine the generalisability of programme theories across borders ( DeSouza, 2016). More recently, it has been used in studies related to understanding:

• how social prescribing interventions operate in the UK’s HE context ( Wallace et al., 2020)
• the effectiveness of feedback interventions in open-ended tasks like essays and reports, identifying the mechanisms that make feedback interventions work ( Ajjawi et al., 2020)
• the communication skills of social work students ( Reith Hall, 2022)
• online faculty development offerings ( Keiller et al., 2022)
• engagement in Synchronous Online Learning ( Mosher et al., 2023)

Together, these studies illustrate the effective use of realist review in HE for unpacking the mechanisms, contextual factors, and outcomes that enable programs to succeed, enabling institutions to create responsive, evidence-based initiatives. Realist review is a suitable method for researching SSP initiatives in HE because these initiatives are complex, context-dependent, and involve multiple stakeholders. SSP initiatives, which aim to engage students in co-creating their learning experiences, are more than one-size-fits-all solutions ( Healey et al., 2014). Their success depends on how they interact with the specific educational contexts (e.g., institutional culture, teaching practices, and student characteristics) and the mechanisms they activate (e.g., student motivation, sense of ownership, and collaboration with faculty). Realist review, with its focus on understanding the "how" and "why" behind outcomes, allows researchers to explore the varied impacts of SSP initiatives across different settings, helping to identify which elements are most effective for specific groups of students or institutional environments ( Pawson & Tilley, 1997; Wong et al., 2013). Using the Context-Mechanism-Outcome (CMO) framework, a realist review can reveal the underlying processes that drive success or failure in these initiatives, providing actionable insights for improving and scaling SSP programs across diverse educational contexts. This protocol paper details the methods we will undertake to complete a realist review of the literature on SSP initiatives in the HE setting.

## Methods

**Figure 1.**
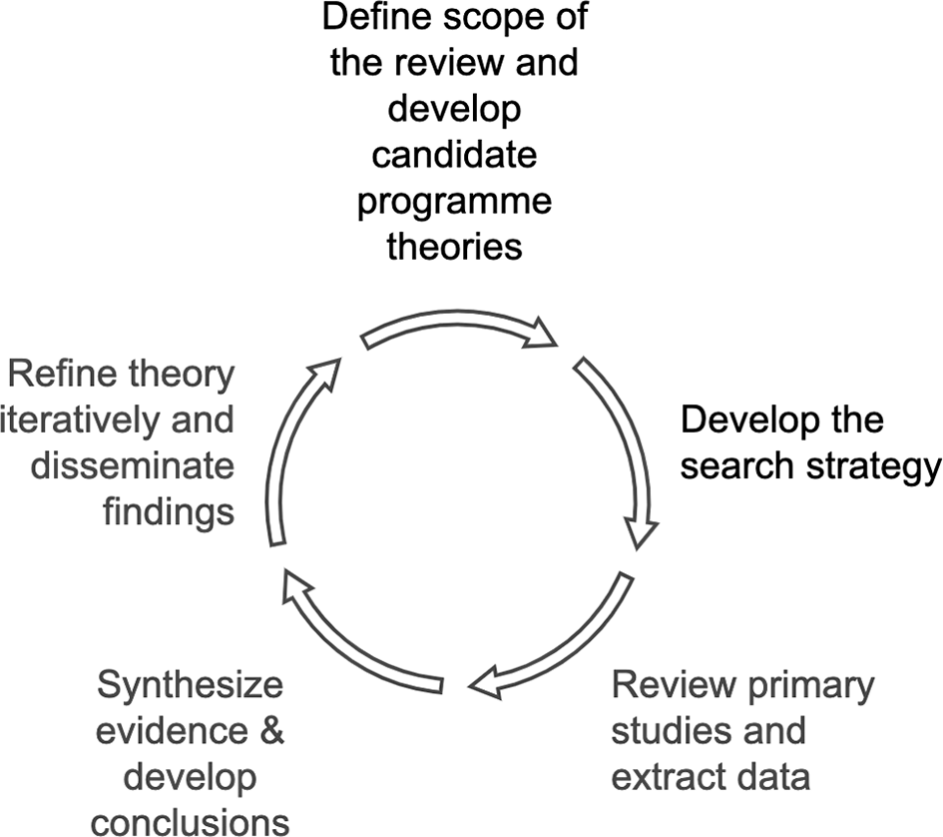
Stages of the Realist Review. The figure has been designed by the authors based on a figure by Teeling et al. (2021) ( https://www.mdpi.com/1660-4601/18/22/11932) and is adapted with full permission from the authors. The corresponding author of this protocol is also the first and corresponding author of the 2021 paper on which Figure 1 is based.

The realist review outlined in this protocol paper will examine the concept of SSP in higher education institutions (HEIs), aiming to identify the underlying mechanisms, contextual factors, and outcomes associated with SSP practices. The study will follow the five-stage methodology ( Figure 1) outlined in the RAMESES (Realist And Meta-narrative Evidence Syntheses: Evolving Standards) guidelines ( Wong et al., 2013).

The realist approach is particularly suitable for this topic, as it seeks to explain *how*, *why*, and *for whom* SSP initiatives work in different HEI settings. We now outline each of the five stages to be undertaken in completing the realist review.

### 1. Define the Scope of the Review and Develop Initial Theories

In a realist review, initial theories are developed by creating candidate programme theories (CPTs). These theories offer a structured approach to examining the causal mechanisms of interventions and how they produce specific outcomes within particular contexts ( Teeling et al., 2021). Therefore, the first step in conducting a realist review is developing CPTs that explain how the designers and implementers of SSP initiatives expect them to work and why ( Pawson & Tilley, 1997).

***Locating key literature***

Before undertaking a realist review, it is necessary to identify key literature to support developing CPT, and we will conduct an initial high-level scoping of the literature ( Kent and Ajjawi, 2022), to facilitate the identification of key literature to map the existing body of research on SSP in HEIs. This review will identify key themes, debates, and gaps in the literature, providing a broad understanding of how SSP has been conceptualised and implemented. The scoping review will use a systematic search strategy to identify relevant studies, reports, and frameworks and will involve conducting a preliminary background search in key databases by searching article titles, abstracts, keywords, and subject headings to guide the development of the CPTs ( Wong et al., 2013). Findings from the scoping review will highlight key contexts, mechanisms, and outcomes (CMOs) reported in the literature and inform CPT development.

***Advisory groups***

Two advisory groups will be convened to provide complementary perspectives and guide the scope of the research and the development of the CPT:

• Local Reference Group/practitioner group: This group will consist of students, educators, and administrators actively involved in SSP initiatives within the study site. Members will be selected using purposive sampling to ensure representation across disciplines, roles, and institutional contexts. This group will contribute lived experiences of SSP practices, offering practical insights into challenges, facilitators, and impacts. Their input will validate emerging theories and help identify relevant real-world contexts ( Teeling et al., 2021).
• Expert Panel: This panel will include researchers, policymakers, and practitioners with expertise in SSP, HE pedagogy, and realist methodologies. The study site has a Teaching and Learning Board with both national and international representation containing this expertise that has agreed to function as the expert panel. The review of the panel will ensure the review’s theoretical and methodological rigour, providing critical feedback on the developing theories and synthesis ( Wong et al., 2013).

Preliminary theories will be developed by synthesising insights from the scoping review and feedback from the advisory groups. These theories will be articulated as CMO configurations ( Pawson, 2013a), hypothesising how specific mechanisms (e.g., shared decision-making, co-creation of curricula) interact with contexts (e.g., institutional culture, disciplinary norms) to produce outcomes (e.g., enhanced student engagement, improved teaching practices). These initial theories will guide data extraction and synthesis in subsequent stages.

### 2. Develop the search strategy

A comprehensive and systematic search strategy will capture a wide range of evidence on SSP in HEIs. The search will cover academic databases, including ERIC, Scopus, ProQuest (social sciences), Web of Science, and PsycINFO, using combinations of keywords and Boolean operators. Databases will be searched from 2014 onwards since the seminal publication on SSP was published in 2014, and the origins of SSP in HEIs in Ireland post-dates that ( Healey et al., 2014; Higher Education Authority, 2016). This will involve an evidence search guided by keywords developed from the CPTs identified in the scoping phase ( Dada et al., 2023). The PCC framework (Population, Concept, Context) is a structured approach used for scoping reviews and systematic searches to define key elements of a research question ( Peters et al., 2020). It is recommended by the Joanna Briggs Institute (JBI) for scoping reviews, helping researchers clearly identify:

• Population (P): Who is the focus of the research?
• Concept (C): What is the main idea, intervention, or phenomenon of interest?
• Context (C): Where or in what setting does the research take place?

The search strategy is illustrated in Table 1.

**Table 1.** Sample Search Strategy for ERIC Database.

Component	Search Terms
**#1 Population**	(“university Student*” OR Undergraduate* OR “first year*” OR freshman* OR sophomore* OR post *graduate* OR graduate* OR “grad student*” OR “post-secondary student*” OR “post secondary student*” OR underclassman OR “Mature student*” OR “senior student*” OR scholar* OR registrant* OR doctora* OR phd* OR master* OR “College Senior*” OR “Two Year College Student*” OR MAINSUBJECT.EXACT.EXPLODE("College Students") OR student NEAR (university OR "third level" OR "higher education"))
**#2 Context**	Universit* OR “third level education” OR college OR “higher education” OR “further education” OR polytechnic* OR “technical college*” OR “technical universit*” OR “Technical Institute*” OR “academic institution*” OR “tertiary education” OR "Postsecondary Education" OR graduat* OR post *graduate* OR fellow* OR “institute of technology” OR MAINSUBJECT.EXACT.EXPLODE("Higher Education") OR MAINSUBJECT.EXACT("Postsecondary Education") OR MAINSUBJECT.EXACT("Fellowships")
**#3 Concept**	“design-based research” OR DBR OR “participatory design” OR PD OR co-creation* OR co-design* OR “student voice*” OR “student role*” OR “student-staff partnership*” OR “student-faculty partnership*” OR “students as partners” OR SAP OR “students as change agents” OR “student engagement” OR “Learner Engagement” OR “student empowerment” OR “student participation” OR “student-staff collaboration*” OR “faculty-student collaboration” OR “Partnership learning communit*” OR Co-learning OR Co-develop* OR Co-research* OR Co-inquiring OR MAINSUBJECT.EXACT("Learner Engagement") OR MAINSUBJECT.EXACT("Student Empowerment") OR MAINSUBJECT.EXACT("Student Participation")
**Final Query**	**#1 AND #2 AND #3**

The PRISMA (Preferred Reporting Items for Systematic Reviews and Meta-Analyses) guidelines will be used to provide a systematic framework for synthesising evidence across various study designs, including observational and interventional research ( Page et al., 2021). While PRISMA is typically associated with systematic reviews with meta-analyses, its adaptability makes it valuable for realist reviews, which focus on exploring the mechanisms and contexts of complex interventions rather than solely assessing effectiveness through controlled trials ( Teeling et al., 2021). The search strategy and findings will be reported ( Teeling et al., 2025) in alignment with PRISMA guidelines ( Page et al., 2021) and the standards for realist reviews ( Wong et al., 2013), ensuring transparency and methodological rigour.

### 3. Review primary studies and extract data

Search results will be independently screened by two reviewers in two stages: (1) title and abstract screening, followed by (2) full-text screening using predefined inclusion and exclusion criteria. This screening will be facilitated by Covidence systematic review software, (2025). an online systematic review management software designed to facilitate the screening, data extraction, and management of literature reviews. In a realist review, it supports the iterative and collaborative nature of screening by enabling reviewers to efficiently apply inclusion and exclusion criteria, track decisions, and manage conflicts ( Teeling et al., 2021). Studies will be included by consensus if they explicitly examine SSP initiatives in HEIs and report relevance to IPT or their constituent CMOC. Only papers that describe SSP initiatives based on the following definition (Adapted from Cook-Sather, Bovill and Felten, 2014, pp. 6-7) will be eligible for inclusion: “a collaborative, reciprocal *process* through which students and faculty or professional staff have the opportunity to contribute equally, although not necessarily in the same ways, to curricular or pedagogical conceptualisation, decision-making, implementation, investigation, or analysis, or in university access and support structures”. The studies selected for inclusion will be critically appraised using the RAMESES quality appraisal criteria ( Wong et al., 2013). This appraisal will ensure that data extraction is focused on studies of sufficient rigour and relevance to the research questions.

To streamline data extraction in alignment with realist review principles, the IPT under investigation will be made explicit by creating and applying custom-designed data extraction forms ( Rycroft Malone et al., 2012). These forms will be tailored to the specific requirements of the realist review, acknowledging that the design of such tools varies depending on the theoretical framework being applied ( Pawson et al., 2004). Standardised forms are often unsuitable for realist reviews due to their theoretical specificity; hence, bespoke forms are developed to capture context-specific insights effectively ( Hunter et al., 2022). These tailored forms will guide extracting and analysing information critical to refining the IPT by identifying contextual factors, mechanisms, and outcomes related to the research questions. The data will be used to collect information on:

• Contexts: Institutional, disciplinary, and cultural factors influencing SSP.
• Mechanisms: Processes, strategies, and interactions driving SSP outcomes.
• Outcomes: Intended and unintended consequences of SSP initiatives.
• Theoretical contributions: Frameworks or models employed in the studies.

Data will be imported to NVivo software (version 14) to organise and manage qualitative data ( Castleberry, 2014). The review will aim to identify evidence that supports, refutes, or refines the initial programme theories.

### 4. Synthesise evidence and develop conclusions

A realist synthesis will be conducted to analyse and integrate the extracted data. This synthesis will focus on identifying patterns in the developing CMOC and refining the IPT. The synthesis process will include:

1. Thematic Analysis: Identifying recurring themes and grouping similar CMOs.
2. Narrative Synthesis: Constructing a coherent account of how SSP initiatives work across different contexts.
3. Refinement of Theories: Comparing emerging patterns with insights from the scoping review and advisory groups to ensure theoretical robustness and practical relevance.

This stage will aim to produce refined theories that explain how SSP initiatives generate their effects, under what conditions, and for whom.

### 5. Refine theory iteratively and disseminate findings

The theories will be refined iteratively, with ongoing input from the local reference group and expert panel. Feedback from these groups will ensure that the findings remain grounded in practical experiences and theoretical rigour.

### 6. Dissemination of findings

Findings will be disseminated through:

• Academic publications in peer-reviewed journals (realist review and realist evaluation).
• Presentations at conferences and seminars.
• Practitioner-oriented outputs, including summaries and guidelines, to inform policy and practice in the study site and more widely in HEI.

Efforts will also be made to produce accessible resources, such as infographics and video summaries, to reach a wider audience of students, educators, and institutional leaders.

## Discussion

This realist review will significantly contribute to the literature on SSP in multiple ways. While SSP has gained increasing attention in recent years, few literature reviews exist in this area, and none have been conducted on the scale of this study. By synthesising the scholarly literature published in the decade following seminal contributions to the field, this review will provide a strong foundation for further research and practice in SSP. It will particularly highlight and develop contemporary research addressing critical factors such as mindset, culture, representation and equity, and the management of disagreement within partnership work ( Mercer-Mapstone & Marie, 2019; Matthews, 2021).

Furthermore, by adopting a research methodology well-established in the health sciences but under-utilised in educational research, this review not only offers a rigorous methodological framework for studying SSP but also demonstrates the potential of realist review for deepening our understanding of the contexts and mechanisms through which educational interventions lead to meaningful outcomes ( Wong et al., 2013; Pawson, 2013a, 2013b). Applying a realist lens in this context will generate nuanced insights into its strengths and limitations when applied to partnership work. Specifically, we will explore which aspects of SSP realist review can effectively illuminate and where it may be less suited to capturing the complex, qualitative, and often intangible dimensions of partnership dynamics ( Felten et al., 2019).

Developing our search strategy has also led us to engage critically with the language and assumptions underpinning SSP. In particular, we have foregrounded the term 'student-staff partnerships' rather than the more commonly used 'Students as Partners' (SAP). The latter implicitly assumes staff participation without fully accounting for the varied roles of faculty and professional staff ( Healey et al., 2014). This distinction is crucial, as it enables us to examine how different staff roles shape partnership experiences and outcomes.

By synthesising existing research, refining methodological approaches, and critically analysing the implications of realist review for SSP, this study will offer valuable contributions to both scholarship and practice. Its findings will inform educators, researchers, and policymakers seeking to foster meaningful and equitable SSP in HE.

## Software availability

This study used Covidence for systematic review management (Covidence, https://www.covidence.org/). A free alternative, **SRDR+ (Systematic Review Data Repository Plus)**, is available at https://srdrplus.ahrq.gov/ and can perform similar functions, including study screening, data extraction, and review management.

## Reporting guidelines

The RAMESES (Realist and Meta-narrative Evidence Synthesis: Evolving Standards) project will inform the Realist Review.

## Author contributions

Teeling, S.P., Lennon, O., McAreavey, N., Farrell, R. Conceptualization, Data Curation, Formal Analysis, Funding Acquisition, Methodology, Validation, Visualization, Writing – Original Draft Preparation, Writing – Review & Editing

## Data Availability

Data availability: No data are associated with this article. Figshare: Exploring the Impact of Student-Staff Partnerships in Higher Education: A Realist Review Protocol. DOI: https://doi.org/10.6084/m9.figshare.28597505 ( Teeling et al., 2025). The project contains the following extended data:
• PRISMA -P checklist Data are available under the terms of the Creative Commons Attribution 4.0 International license (CC-BY 4.0).
