# Exploring the Impact of Student-Staff Partnerships in Higher Education: A Realist Review Protocol.

**DOI:** 10.12688/f1000research.163068.2

**Published:** 2026-01-27

**Authors:** Seán Paul Teeling, Naomi McAreavey, Rachel Farrell, Olive Lennon

**Affiliations:** 1 University College Dublin School of Nursing Midwifery and Health Systems, Dublin, Leinster, Ireland; 2 University College Dublin School of English Drama and Film, Dublin, Leinster, Ireland; 3 University College Dublin School of Education, Dublin, Leinster, Ireland; 4 University College Dublin School of Public Health Physiotherapy and Sports Science, Dublin, Leinster, Ireland

**Keywords:** Student-staff partnership, higher education, realist review, programme theory, engagement strategies, context-mechanism-outcome, institutional change, policy development

## Abstract

**Background:**

Student-staff partnership (SSP) in higher education (HE) is rooted in democratic education and critical pedagogy, advocating for active student engagement. While widely recognised for fostering student success, SSP initiatives vary in definition, implementation, and impact. In Ireland, national policy has positioned SSP as central to student engagement, yet there is limited synthesis of the contextual factors and mechanisms influencing its success. Existing literature highlights benefits such as increased engagement and enhanced learning but often overlooks challenges, including power dynamics, resistance to change, and inconsistent institutional commitment.

**Aim:**

This protocol outlines the methodology for a realist review, forming the first stage of a broader realist inquiry into SSP initiatives. The review will synthesise existing evidence to understand how, why, and in what contexts SSP initiatives succeed or face challenges in HE. Specifically, it will identify the contextual factors, mindsets, and engagement strategies that enable meaningful partnerships, as well as mechanisms for overcoming barriers. The review will generate initial programme theories (IPTs) to inform a subsequent realist evaluation within University College Dublin (UCD).

**Method:**

A realist review methodology will be used to explore the interactions between context, mechanisms, and outcomes in SSP initiatives. The study will follow a five-stage realist review process: scoping the literature, developing IPTs, systematically reviewing evidence, synthesising findings, and refining theories with expert input. The Context-Mechanism-Outcome Configuration (CMOC) framework will guide the analysis.

**Conclusion:**

This protocol sets out the approach for developing evidence-informed programme theories on SSP. These theories will underpin a subsequent realist evaluation within UCD to refine SSP implementation strategies. Findings will inform institutional strategies, policy development, and academic practice, with dissemination through academic and practitioner-focused outputs.

## Background

The concept of student–staff partnership (SSP) is rooted in advocacy for more democratic approaches to education and critical pedagogy ( Bovill, 2012). This has significantly influenced contemporary understandings of SSP ( Bovill, 2019, p. 6). Although SSP is also frequently discussed as “Students as Partners” (SaP), we use SSP in this study because it explicitly recognises both parties in the partnership ( Cook-Sather et al., 2018). The emergence of SSP marked a departure from consumeristic and neo-liberal educational paradigms, prioritising democratic values and social justice ( Lubicz-Nawrocka and Bao, 2025).

A key challenge in advancing SSP scholarship and practice is that closely related initiatives are described using a range of partially overlapping terms. In addition to “student–staff partnership” and “students as partners,” the literature frequently uses terms such as co-creation, co-design, participatory design, student voice, and students as change agents. While these concepts are not always identical in purpose or depth of shared influence, they often describe initiatives in which students contribute to the conceptualisation, design, implementation, evaluation, or governance of learning and teaching and/or broader institutional practices. Reflecting this, the literature reports both a broad consensus regarding the potential value of partnership and variability in how central values and concepts are defined across publications ( Mercer-Mapstone et al., 2017), alongside the importance of careful naming and conceptual clarity in partnership practices ( Casey, 2024).

In this review, partnership is understood as a relational and process-oriented practice. A widely accepted definition articulated by Bovill, Cook-Sather, and Felten (2016) describes partnership as a “collaborative, reciprocal process through which all participants have the opportunity to contribute equally, [although not necessarily in the same ways] to curricular or pedagogical conceptualisation, decision-making, implementation, investigation, or analysis” (pp. 6–7). In higher education, this is often enacted through practices such as students partnering with staff to co-design modules or assessments, acting as student consultants who provide structured feedback on learning and teaching, serving on partnership committees or curriculum working groups, and engaging in co-inquiry projects where students and staff jointly investigate and improve aspects of the learning environment. While this definition usefully captures many partnership practices, it may overlook other forms of partnership, including those between students and non-academic/professional staff members or in areas such as civic engagement, research, and the enhancement of the wider student experience. Within partnership literature, such non-academic partnerships are notably underrepresented ( Healey, Flint, and Harrington, 2014; Mercer-Mapstone et al., 2017). To avoid overly prescriptive definitions regarding the outcomes or foci of partnership practices, Healey, Flint, and Harrington (2014) advocate for an open-ended understanding of partnership as a “process” of student engagement in higher education rather than merely an outcome or product (p. 14). In addition, partnership scholarship cautions that initiatives labelled as “partnership” may vary in authenticity and the extent to which students exert meaningful influence ( Barradell & Bell, 2021), reinforcing the importance of attending to what constitutes genuine partnership in practice ( Casey, 2024). Accordingly, “meaningful partnership” in this review is operationalised as evidence of reciprocal contribution and student influence in decision-making beyond consultation, consistent with this SSP definition and the partnership authenticity criteria used at full-text screening and extraction.

Existing literature reports a range of positive outcomes associated with SSP. Reported benefits for students and staff who directly participate in partnership work include enhanced engagement and belonging, strengthened relationships and collaboration, increased confidence and agency, and improvements in learning-related motivation and metacognitive engagement ( Healey, Flint, and Harrington, 2014; Mercer-Mapstone et al., 2017; Felten et al., 2019). Partnership is also positioned as a route to improved educational design, relevance, and inclusivity—benefits that may extend beyond those directly involved to indirect beneficiaries who experience the resulting changes ( Healey, Flint, and Harrington, 2014; Mercer-Mapstone & Marie, 2019). At the same time, outcomes may be uneven, absent, mixed, or contradictory across stakeholder groups, depending on how partnership is enacted and supported.

Alongside these benefits, challenges and strategies for addressing them are also documented, including power dynamics and role ambiguity, resistance to change, navigating institutional norms, ensuring inclusivity and representation, managing disagreement, and sustaining partnership work over time ( Bovill et al., 2016; Abbot & Cook, 2020; Bindra et al., 2018; Carroll, 2023). More recent scholarship emphasises the importance of partnership readiness and mindset ( Gauthier, 2020; Rideau, 2022), cultural context and inclusive classroom practices ( Cook-Sather et al., 2020), and equity and epistemic justice within partnership work ( Bindra et al., 2018; De Bie et al., 2021). In medical education, a recent overview further consolidates challenges and practical guidance across learner, teacher, and institutional perspectives ( Könings et al., 2021), underscoring that challenges and their management are not a gap in the broader literature, but rather an important body of knowledge to be integrated into theory-building for SSP.

While the literature provides rich descriptions of partnership activities, benefits, and challenges, what remains less well understood is how SSP produces outcomes in different settings—through what mechanisms, triggered in what contexts, and with what consequences for different stakeholder groups, including direct participants and indirect beneficiaries. In keeping with partnership scholarship that frames SSP as a relational process underpinned by an ethic of reciprocity, we conceptualise SSP as a values-based social practice that is enacted differently across contexts, rather than as a bounded intervention ( Cook-Sather et al., 2014; Bovill et al., 2016). SSP practices are complex and context-dependent, and are more than one-size-fits-all solutions ( Healey, Flint, and Harrington, 2014). A realist review is therefore well suited to synthesising this evidence because it aims to explain how complex social practices and programmes unfold (or fail to unfold as intended) by developing and refining context–mechanism–outcome configurations (CMOCs) that account for variation across settings and stakeholders ( Pawson & Tilley, 1997; Wong et al., 2013; Greenhalgh et al., 2017).

## Study objectives and location

This study is being undertaken by a team of four fellows in teaching and academic development at University College Dublin (UCD). UCD is Ireland’s largest university and a leading global institution. It hosts over 38,000 students from 152 countries and is ranked among the top 1% of HEIs worldwide. It offers diverse disciplines across six colleges, including Arts and Humanities, Social Sciences and Law, and Health and Agricultural Sciences. The fellows come from three of the six colleges, representing the disciplines of Education, English, Nursing, and Physiotherapy. Student partnership is central to UCD’s strategy to 2030, and the institution set the theme for our fellowship project, which it sponsors ( University College Dublin, 2024).

This realist review is international in scope and forms the first stage of a broader realist inquiry. The purpose of the review is to develop and refine initial programme theories that explain how student–staff partnership (SSP) approaches in higher education generate outcomes—through what mechanisms, in what contexts, and for whom. In keeping with partnership scholarship that frames SSP as a relational, values-based social practice, we do not treat SSP as a standardised intervention; instead, we focus on how partnership approaches are enacted differently across institutional and disciplinary settings. The review will explicitly differentiate outcomes for (a) direct participants in partnership activity (students, academic staff, and professional staff) and (b) indirect beneficiaries affected by partnership-informed changes (e.g., cohorts or staff groups who experience changes to educational design, supports, or practices).

Our research questions are:

1. What contextual factors (including institutional culture, structures, roles, and resources) enable or constrain meaningful SSP initiatives in higher education, and how do these contexts shape outcomes for direct participants and indirect beneficiaries?
2. How do participant characteristics (e.g., partnership readiness, beliefs about roles/power, prior experience) and SSP approaches (e.g., co-design, co-inquiry, governance partnership, participatory design) shape engagement, the navigation of barriers, and outcomes of SSP in different contexts?
3. What mechanisms and strategies support the effective management of common SSP challenges (e.g., power dynamics, resistance, inclusivity, workload, recognition), and what consequences follow for direct participants and indirect beneficiaries?

The resulting programme theories will inform a subsequent realist evaluation within the UCD setting, supporting the development of a well-defined, inclusive, and context-sensitive model for SSP that can be adapted and applied in other higher education contexts.

## Methods

### Realist inquiry

We have adopted realist inquiry as the method for this study because it effectively unpacks the mechanisms of complex systems, providing actionable insights that benefit all stakeholders ( Wong et al., 2013). Realist inquiry is a methodology designed to understand complex interventions by exploring the “how” and “why” behind outcomes in specific contexts ( Wong et al., 2017). Unlike traditional evaluation approaches that focus on whether interventions work, realist inquiry delves deeper into the mechanisms that produce outcomes, considering the influence of various contexts ( Teeling et al., 2021). It is beneficial for evaluating interventions in dynamic, multifaceted settings, such as healthcare, where multiple factors influence results ( Pawson, 2013a; Wong et al., 2013; Manzano et al., 2017).

A fundamental principle of realist inquiry is that interventions do not work in a vacuum. Their success or failure is not merely a result of the intervention itself. However, it is significantly influenced by the context in which they are implemented and the mechanisms activated within that context ( Wong et al., 2017). This approach emphasises understanding *why* and *how* interventions work, offering a more nuanced understanding than simple cause-effect evaluations ( Pawson & Tilley, 1997).

Realist inquiry consists of two main components: realist review and realist evaluation, each serving distinct but complementary roles ( Teeling et al., 2021). Realist reviews are used to synthesise existing evidence and develop programme theories, while realist evaluations test and refine these theories in specific settings ( Pawson et al., 2005; Marchal, 2012). Together, they offer a comprehensive framework for understanding interventions' complexities and outcomes. This protocol paper outlines the rationale and methods for using realist review to explore the complex dynamics of SSP in HE.

### Realist review

Realist review (also referred to as realist synthesis) is a theory-driven approach for synthesising evidence from diverse study designs to develop and refine explanations about how and why outcomes occur in different settings ( Pawson et al., 2005; Wong et al., 2013). Realist review aims to generate explanatory insights that can inform practice and policy by identifying the mechanisms through which outcomes are generated (or not), and the contextual conditions that shape those mechanisms ( Wong et al., 2013).

A core analytic tool in realist review is the Context–Mechanism–Outcome Configuration (CMOC) framework, which describes how outcomes arise when particular mechanisms are triggered in particular contexts ( Pawson, 2013b; Wong et al., 2013). In this review:

• Context (C) refers to the conditions, settings, or environments in which SSP is enacted, including organisational structures, cultural norms, policies, resources, and stakeholder relationships. Context can facilitate or constrain mechanisms and therefore shape outcomes ( Manzano, 2016; Pawson, 2013a).
• Mechanism (M) refers to the underlying causal processes that explain how outcomes are generated. Mechanisms are often not directly observable; they can be inferred from how people respond to the resources, opportunities, and constraints present in context. For example, SSP may trigger mechanisms such as a sense of legitimacy, ownership, reciprocity, or responsibility, which in turn shape outcomes ( Pawson & Tilley, 1997; Wong et al., 2013).
• Outcome (O) refers to the consequences of SSP approaches, including intended and unintended effects, which can vary by context and by the mechanisms that are activated ( Greenhalgh et al., 2017; Pawson, 2013a, 2013b).

For example, in a context where departmental culture supports shared governance and time is formally allocated for partnership work (C), involving students as genuine decision-makers in assessment design may trigger a greater sense of agency, legitimacy, and trust among student and staff partners (M), leading to stronger engagement and clearer assessment expectations for direct participants, and potentially improved learning design quality for the wider cohort (O). In less supportive contexts (C), similar activities may trigger perceptions of tokenism, risk, or reduced ownership (M), resulting in weaker, absent, or contradictory outcomes across stakeholder groups (O).

Consistent with the aims of this review, outcomes will be examined separately for (a) direct participants in SSP activity (students and staff who participate) and (b) indirect beneficiaries affected by partnership-informed changes (e.g., cohorts or staff groups experiencing changes in educational design, supports, or practices). Outcomes may be absent, mixed, or even contradictory across stakeholder groups depending on how SSP is enacted and supported.

Combining these elements into CMOCs enables a deeper explanatory account of SSP by identifying patterns in what tends to work, for whom, and under what conditions ( Pawson, 2013a). Realist review also serves as a foundational step for realist evaluation by developing programme theories: testable hypotheses about the relationships between contexts, mechanisms, and outcomes. These programme theories can then be refined through subsequent empirical research, such as realist evaluation ( Pawson, 2013a; Pawson et al., 2005).

### The use of realist review in higher education

Realist review is emerging as a valuable approach in HE for studying complex educational programmes and practices where outcomes are contingent on contextual conditions and stakeholder responses. Educational theories that explain the application, interpretation, and purpose of learning underpin teaching and learning activities ( Artino & Konopasky, 2018). These theoretical concepts help explain learning processes, inform educational approaches, curricula, and assessments ( Khalil & Elkhider, 2016), and enable evaluation of approaches to teaching ( Albert et al., 2007; Rees & Monrouxe, 2010). While systematic reviews have examined the concept of SSP in teaching and learning ( Mercer-Mapstone et al., 2017) and developed theorisation related to partnership praxis ( Matthews et al., 2018), these syntheses often summarise outcomes and enabling practices but do not consistently develop and test explanatory programme theories that account for why similar SSP approaches produce different outcomes across contexts and stakeholder groups. In addition, definitional variation and the breadth of SSP purposes and forms can limit the transferability of generic recommendations. Building on this foundation, this review will explicate how outcomes are generated through mechanisms operating in specific contexts and for whom (direct and indirect stakeholders). A broader, theory-driven design is required to explain how and why SSP approaches generate outcomes and under what conditions they are more or less likely to do so ( Suri & Clarke, 2009; Wong et al., 2012). Realist review is particularly suited to this purpose because it seeks to explain to what extent, how, why, for whom, and in what circumstances complex educational programmes and practices generate outcomes.

Realist review in education has previously been used to develop programme theory related to factors affecting teaching and learning for medicines supply management training ( Brown et al., 2013), to study how structural and cultural elements combine to build a “quality culture” in HE ( Bendermacher et al., 2017), and to examine the generalisability of programme theories across borders ( DeSouza, 2016). More recently, realist reviews have been applied to topics such as how social prescribing approaches operate in the UK’s HE context ( Wallace et al., 2020), how feedback processes function in open-ended tasks such as essays and reports ( Ajjawi et al., 2020), the communication skills of social work students ( Reith Hall, 2022), online faculty development offerings ( Keiller et al., 2022), and engagement in synchronous online learning ( Mosher et al., 2023).

Together, these studies illustrate the potential of realist review in HE for unpacking the mechanisms, contextual factors, and outcomes that enable programmes and practices to succeed or falter, supporting institutions to develop responsive, evidence-informed initiatives. Realist review is a suitable method for researching SSP approaches in HE because these approaches are complex, context-dependent, and involve multiple stakeholders. SSP approaches, which aim to engage students in co-creating aspects of their learning experiences, are more than one-size-fits-all solutions ( Healey et al., 2014). Their outcomes depend on how they interact with specific educational contexts (e.g., institutional culture, teaching practices, and student characteristics) and the mechanisms they trigger (e.g., motivation, sense of ownership, reciprocity, and collaboration). By using CMOCs, a realist review can reveal how and why SSP approaches generate different outcomes across settings and stakeholder groups, providing explanatory insights that can inform improvement and adaptation across diverse HE contexts ( Pawson & Tilley, 1997; Wong et al., 2013). This protocol paper details the methods we will undertake to complete a realist review of the literature on SSP approaches in HE.

### Irish policy context for subsequent realist evaluation

Although the realist review is international in scope, it forms the theory-building phase of a wider inquiry that will include a subsequent realist evaluation in an Irish university setting. For transparency about this broader programme of work, we report relevant Irish policy context here (rather than in the Background), and treat it as a contextual layer in later theory testing and refinement.

In Ireland, the origins of SSP can be traced to the Higher Education Authority (HEA) report Enhancing Student Engagement in Decision-Making (2016), which promoted students as partners in Irish higher education rather than treating them as consumers or passive recipients, and outlined principles for strengthening student–staff engagement and partnership in institutional decision-making. Subsequently, the National Student Engagement Programme has been instrumental in translating this vision into action nationally ( Higher Education Authority, 2021). “Student success” was one of four strategic priorities of the National Forum for the Enhancement of Teaching and Learning in Higher Education (2019–2021), with its vision of success developed in partnership with students ( National Forum, 2019). The National Forum came under the aegis of the HEA in January 2022, and the HEA’s Student Engagement and Teaching & Learning Committee was established thereafter. In this sense, SSP remains at the heart of national higher education policy in Ireland.

SSP has been increasingly adopted across Irish HEIs, with published examples spanning teacher education and institution-level partnership initiatives. Brennan (2021), for example, describes partnership work in Irish university-based teacher education through the Student Teacher Educational Research (STER) project, highlighting both pedagogical benefits and implementation challenges. Munro et al. (2022) document a student–staff partnership initiative at Maynooth University and Maynooth Students’ Union, illustrating how partnership can support institution-level development work while also surfacing practical challenges and learning for participants. Together, these studies suggest growing activity and institutional interest, while also underscoring the need for context-sensitive analysis rather than assumptions of uniform sector-wide implementation. More broadly, institutions in Ireland and internationally have embraced SSP programmes, values, and objectives to varying degrees. While the literature suggests a general consensus regarding the benefits of partnership, there remains variability in how central values and concepts are defined across publications ( Mercer-Mapstone et al., 2017). This review will therefore attend to how policy and institutional conditions shape the enactment of SSP and the mechanisms and outcomes that follow across different contexts.

## Methods

**Figure 1.**
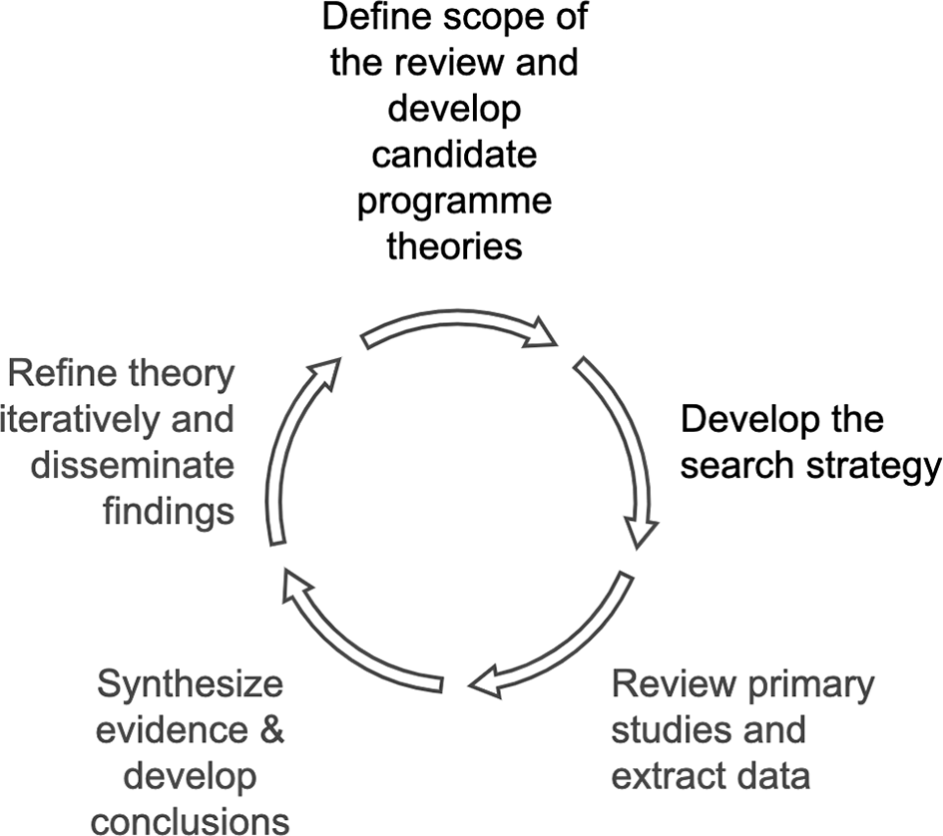
Stages of the Realist Review. The figure has been designed by the authors based on a figure by Teeling et al. (2021) ( https://www.mdpi.com/1660-4601/18/22/11932) and is adapted with full permission from the authors. The corresponding author of this protocol is also the first and corresponding author of the 2021 paper on which Figure 1 is based.

The realist review outlined in this protocol paper will examine the concept of SSP in higher education institutions (HEIs), aiming to identify the underlying mechanisms, contextual factors, and outcomes associated with SSP practices. The study will follow the five-stage methodology ( Figure 1) outlined in the RAMESES (Realist And Meta-narrative Evidence Syntheses: Evolving Standards) guidelines ( Wong et al., 2013).

The realist approach is particularly suitable for this topic, as it seeks to explain *how*, *why*, and *for whom* SSP initiatives work in different HEI settings. We now outline each of the five stages to be undertaken in completing the realist review.

In line with realist principles of generative causation, initial theories will be developed by creating candidate programme theories (CPTs). These theories provide a structured way to explain how SSP practices generate outcomes for different stakeholders in particular contexts, focusing on generative mechanisms rather than assuming linear cause–effect relationships ( Pawson & Tilley, 1997; Wong et al., 2013). Therefore, the first step in conducting this realist review is developing CPTs that articulate how SSP designers and implementers expect partnership to work, for whom, and under what conditions ( Pawson & Tilley, 1997).

***Locating key literature***

Before undertaking a realist review, it is necessary to identify key literature to support developing CPT, and we will conduct an initial high-level scoping of the literature ( Kent and Ajjawi, 2022), to facilitate the identification of key literature to map the existing body of research on SSP in HEIs. This review will identify key themes, debates, and gaps in the literature, providing a broad understanding of how SSP has been conceptualised and implemented. The scoping review will use a systematic search strategy to identify relevant studies, reports, and frameworks and will involve conducting a preliminary background search in key databases by searching article titles, abstracts, keywords, and subject headings to guide the development of the CPTs ( Wong et al., 2013). Findings from the scoping review will highlight key contexts, mechanisms, and outcomes (CMOs) reported in the literature and inform CPT development.

***Advisory groups***

Two advisory groups will be convened to provide complementary perspectives and guide the scope of the research and the development of the CPT:

• Local Reference Group/practitioner group: This group will consist of students, educators, and administrators actively involved in SSP initiatives within the study site. Members will be selected using purposive sampling to ensure representation across disciplines, roles, and institutional contexts. This group will contribute lived experiences of SSP practices, offering practical insights into challenges, facilitators, and impacts. Their input will validate emerging theories and help identify relevant real-world contexts ( Teeling et al., 2021).
• Expert Panel: This panel will include researchers, policymakers, and practitioners with expertise in SSP, HE pedagogy, and realist methodologies. The study site has a Teaching and Learning Board with both national and international representation containing this expertise that has agreed to function as the expert panel. The review of the panel will ensure the review’s theoretical and methodological rigour, providing critical feedback on the developing theories and synthesis ( Wong et al., 2013).

Preliminary theories will be developed from the exploratory scoping phase described in Stage 1 (an internally conducted scoping search to identify seminal and conceptually rich sources), together with advisory group input. These theories will be articulated as CMO configurations ( Pawson, 2013a), hypothesising how specific mechanisms (e.g., shared decision-making, co-creation of curricula) interact with contexts (e.g., institutional culture, disciplinary norms) to produce outcomes (e.g., enhanced student engagement, improved teaching practices). These initial theories will guide data extraction and synthesis in subsequent stages.

### Develop the search strategy

A comprehensive and systematic search strategy will capture a wide range of evidence on SSP in HEIs. The search will cover academic databases, including ERIC, Scopus, ProQuest (social sciences), Web of Science, and PsycINFO, using combinations of keywords and Boolean operators. As some SSP-focused journals may be inconsistently indexed in major databases, database searching will be supplemented by targeted hand-searching of key SSP journals and their websites (e.g., International Journal for Students as Partners, Student Engagement in Higher Education Journal, Journal of Educational Innovation, Partnership and Change). Databases will be searched from 2014 onwards, reflecting the consolidation of Students as Partners as a named field following the publication of a seminal text in 2014 and the subsequent growth in SSP-focused scholarship ( Healey et al., 2014). To ensure foundational and conceptually relevant work published prior to 2014 is not missed (including studies using adjacent terminology), we will use forward and backward citation chasing/“pearl growing” ( Haddaway et al., 2022) from seminal papers and key reviews to identify earlier influential sources where relevant to theory development. The search will be guided by keywords developed from the CPTs identified in the scoping phase ( Dada et al., 2023). The PCC framework (Population, Concept, Context) is a structured approach used for scoping reviews and systematic searches to define key elements of a research question ( Peters et al., 2020). It is recommended by the Joanna Briggs Institute (JBI) for scoping reviews, helping researchers clearly identify:

• Population (P): Who is the focus of the research?
• Concept (C): What is the main idea, intervention, or phenomenon of interest?
• Context (C): Where or in what setting does the research take place?

The search strategy is illustrated in Table 1.

**Table 1.** Sample Search Strategy for ERIC Database.

Component	Search Terms
**#1 Population**	(“university Student*” OR Undergraduate* OR “first year*” OR freshman* OR sophomore* OR post *graduate* OR graduate* OR “grad student*” OR “post-secondary student*” OR “post secondary student*” OR underclassman OR “Mature student*” OR “senior student*” OR scholar* OR registrant* OR doctora* OR phd* OR master* OR “College Senior*” OR “Two Year College Student*” OR MAINSUBJECT.EXACT.EXPLODE("College Students") OR student NEAR (university OR "third level" OR "higher education"))
**#2 Context**	Universit* OR “third level education” OR college OR “higher education” OR “further education” OR polytechnic* OR “technical*” OR “technical universit*” OR “Technical Institute*” OR “academic institution*” OR “tertiary education” OR "Postsecondary Education" OR graduat* OR post *graduate* OR fellow* OR “institute of technology” OR MAINSUBJECT.EXACT.EXPLODE("Higher Education") OR MAINSUBJECT.EXACT("Postsecondary Education") OR MAINSUBJECT.EXACT("Fellowships")
**#3 Concept**	“design-based research” OR DBR OR “participatory design” OR PD OR co-creation* OR co-design* OR “student voice*” OR “student role*” OR “student-staff partnership*” OR “student-faculty partnership*” OR “students as partners” OR SAP OR “students as change agents” OR “student engagement” OR “Learner Engagement” OR “student empowerment” OR “student participation” OR “student-staff collaboration*” OR “faculty-student collaboration” OR “Partnership learning communit*” OR Co-learning OR Co-develop* OR Co-research* OR Co-inquiring OR MAINSUBJECT.EXACT("Learner Engagement") OR MAINSUBJECT.EXACT("Student Empowerment") OR MAINSUBJECT.EXACT("Student Participation")
**Final Query**	**#1 AND #2 AND #3**

The PRISMA (Preferred Reporting Items for Systematic Reviews and Meta-Analyses) guidelines will be used to support transparent reporting of the search process and study selection, including documentation of screening decisions and presentation of a PRISMA flow diagram ( Page et al., 2021). Realist review conduct, synthesis and reporting will be guided by RAMESES standards, which are specific to theory-driven evidence synthesis and the development and refinement of Context–Mechanism–Outcome configurations ( Wong et al., 2013). The search strategy and study selection will be reported in alignment with PRISMA ( Page et al., 2021), and the overall review will be reported in accordance with RAMESES guidance to ensure transparency and methodological rigour ( Wong et al., 2013; Teeling et al., 2021, 2025).

### Review primary studies and extract data

Search results will be independently screened by two reviewers in two stages: (1) title and abstract screening, followed by (2) full-text screening using predefined inclusion and exclusion criteria. This screening will be facilitated by Covidence systematic review software (2025) an online systematic review management software designed to facilitate the screening, data extraction, and management of literature reviews. In a realist review, it supports the iterative and collaborative nature of screening by enabling reviewers to efficiently apply inclusion and exclusion criteria, track decisions, and manage conflicts ( Teeling et al., 2021). Studies will be included by consensus if they explicitly examine SSP initiatives in HEIs and report relevance to IPT or their constituent CMOC. To distinguish meaningful partnerships from pseudo-partnerships, full-text screening will include an explicit “partnership authenticity” assessment using predefined criteria (e.g., evidence of reciprocal contribution, shared decision-making influence, and student involvement beyond consultation). Where necessary, disagreements will be resolved through discussion to reach consensus. Studies will be categorised as partnership/partial partnership/consultative involvement, and this categorisation will be used both to support inclusion decisions and to interpret CMOCs (including explaining mixed or negative outcomes). All potentially relevant papers will be assessed against the following SSP definition (Adapted from Cook-Sather, Bovill and Felten, 2014, pp. 6–7) and categorised according to the degree of partnership: “a collaborative, reciprocal *process* through which students and faculty or professional staff have the opportunity to contribute equally, although not necessarily in the same ways, to curricular or pedagogical conceptualisation, decision-making, implementation, investigation, or analysis, or in university access and support structures”. The studies selected for inclusion will be critically appraised using the RAMESES quality appraisal criteria ( Wong et al., 2013). This appraisal will ensure that data extraction is focused on studies of sufficient rigour and relevance to the research questions.

To streamline data extraction in alignment with realist review principles, the IPT under investigation will be made explicit by creating and applying custom-designed data extraction forms ( Rycroft Malone et al., 2012). These forms will be tailored to the specific requirements of the realist review, acknowledging that the design of such tools varies depending on the theoretical framework being applied ( Pawson et al., 2004). Standardised forms are often unsuitable for realist reviews due to their theoretical specificity; hence, bespoke forms are developed to capture context-specific insights effectively ( Hunter et al., 2022). These tailored forms will guide extracting and analysing information critical to refining the IPT by identifying contextual factors, mechanisms, and outcomes related to the research questions. The data will be used to collect information on:

• Contexts: Institutional, disciplinary, and cultural factors influencing SSP.
• Mechanisms: Processes, strategies, and interactions driving SSP outcomes.
• Direct participants (who?): The stakeholders directly involved in the SSP initiative (e.g., students, staff) and relevant characteristics (e.g., role, discipline, level/year where reported).
• Indirect beneficiaries (who?): Individuals or groups not directly participating but affected by SSP-related changes (e.g., wider student cohorts, module/class groups, departments).
• Outcomes - direct participants: Intended and unintended consequences for those directly involved in SSP initiatives.
• Outcomes - indirect beneficiaries: Intended and unintended consequences for those indirectly affected by SSP-related changes.
• Contradictory/mixed outcomes across groups (yes/no + notes): Whether outcomes differ between direct participants and indirect beneficiaries, including brief notes and supporting evidence where reported.
• Theoretical contributions: Frameworks or models employed in the studies.
• Degree of partnership: Authenticity category (partnership/partial partnership/consultative involvement) with a brief justification excerpt and page reference.

Data will be imported to NVivo software (version 14) to organise and manage qualitative data ( Castleberry, 2014). The review will aim to identify evidence that supports, refutes, or refines the initial programme theories.

### Synthesise evidence and develop conclusions

A realist synthesis will be conducted to analyse and integrate the extracted data. This synthesis will focus on identifying patterns in the developing CMOC and refining the IPT. To ensure outcomes are interpreted in relation to who is affected, CMOCs will be developed and reported with explicit “for whom” tags, distinguishing between direct participants in SSP initiatives and indirect beneficiaries affected by partnership-led changes. Where reported, contradictory or mixed outcomes across these groups will be examined as part of theory refinement. The synthesis process will include:

1. Thematic Analysis: Identifying recurring themes and grouping similar CMOCs, including patterns that differentiate effects for direct participants versus indirect beneficiaries.
2. Narrative Synthesis: Constructing a coherent account of how SSP initiatives work across different contexts, and for whom, by integrating CMOCs that are separately developed or explicitly labelled according to whether outcomes relate to direct participants or indirect beneficiaries.
3. Refinement of Theories: Comparing emerging patterns with insights from the scoping review and advisory groups to ensure theoretical robustness and practical relevance, including consideration of cases where partnership benefits accrue primarily to direct participants, are absent for indirect beneficiaries, or diverge across groups.

This stage will aim to produce refined theories that explain how SSP initiatives generate their effects, under what conditions, and for whom.

### Refine theory iteratively and disseminate findings

The theories will be refined iteratively, with ongoing input from the local reference group and expert panel. Feedback from these groups will ensure that the findings remain grounded in practical experiences and theoretical rigour.

### Dissemination of findings

The theories will be refined iteratively, with ongoing input from the local reference group and expert panel. Feedback from these groups will ensure that the findings remain grounded in practical experiences and theoretical rigour. Findings will be disseminated through:

• Academic publications in peer-reviewed journals (realist review and realist evaluation).
• Presentations at conferences and seminars.
• Practitioner-oriented outputs, including summaries and guidelines, to inform policy and practice in the study site and more widely in HEI.

Efforts will also be made to produce accessible resources, such as infographics and video summaries, to reach a wider audience of students, educators, and institutional leaders.

## Discussion

This realist review will significantly contribute to the literature on SSP in multiple ways. While SSP has gained increasing attention in recent years, few literature reviews exist in this area, and none have been conducted on the scale of this study. By synthesising the scholarly literature published in the decade following seminal contributions to the field, this review will provide a strong foundation for further research and practice in SSP. It will particularly highlight and develop contemporary research addressing critical factors such as mindset, culture, representation and equity, and the management of disagreement within partnership work ( Mercer-Mapstone & Marie, 2019; Matthews, 2021).

Furthermore, by adopting a research methodology well-established in the health sciences but under-utilised in educational research, this review not only offers a rigorous methodological framework for studying SSP but also demonstrates the potential of realist review for deepening our understanding of the contexts and mechanisms through which educational initiatives lead to meaningful outcomes ( Wong et al., 2013; Pawson, 2013a, 2013b). Applying a realist lens in this context will generate nuanced insights into its strengths and limitations when applied to partnership work. Specifically, we will explore which aspects of SSP realist review can effectively illuminate and where it may be less suited to capturing the complex, qualitative, and often intangible dimensions of partnership dynamics ( Felten et al., 2019).

Developing our search strategy has also led us to engage critically with the language and assumptions underpinning SSP. In particular, we have foregrounded the term 'student-staff partnerships' rather than the more commonly used 'Students as Partners' (SAP). The latter implicitly assumes staff participation without fully accounting for the varied roles of faculty and professional staff ( Healey et al., 2014). This distinction is crucial, as it enables us to examine how different staff roles shape partnership experiences and outcomes.

By synthesising existing research, refining methodological approaches, and critically analysing the implications of realist review for SSP, this study will offer valuable contributions to both scholarship and practice. Its findings will inform educators, researchers, and policymakers seeking to foster meaningful and equitable SSP in HE.

## Software availability

This study used Covidence for systematic review management (Covidence, https://www.covidence.org/). A free alternative, **SRDR+ (Systematic Review Data Repository Plus)**, is available at https://srdrplus.ahrq.gov/ and can perform similar functions, including study screening, data extraction, and review management.

## Reporting guidelines

The RAMESES (Realist and Meta-narrative Evidence Synthesis: Evolving Standards) project will inform the Realist Review.

## Author contributions

Teeling, S.P., Lennon, O., McAreavey, N., Farrell, R. Conceptualization, Data Curation, Formal Analysis, Funding Acquisition, Methodology, Validation, Visualization, Writing – Original Draft Preparation, Writing – Review & Editing.

## Data Availability

Data availability: No data are associated with this article. Figshare: Exploring the Impact of Student-Staff Partnerships in Higher Education: A Realist Review Protocol. DOI: https://doi.org/10.6084/m9.figshare.28597505 ( Teeling et al., 2025). The project contains the following extended data:
• PRISMA -P checklist Data are available under the terms of the Creative Commons Attribution 4.0 International license (CC-BY 4.0).
