# Supplementary material for: Exploring the Impact of Student-Staff Partnerships in Higher Education: A Realist Review Protocol
Source: F1000Res. 2026 Jan 27;14:409. Originally published 2025 Apr 7. [Version 2] doi: 10.12688/f1000research.163068.2 (PMC12891955; doi:10.12688/f1000research.163068.2)
Supplement: Peer review history

# Reviewer report: Reviewer response for version 2

**DOI:** 10.5256/f1000research.194854.r453383

**Published:** 2026-02-10

**Authors:** Karen D Könings

**Affiliations:** 1 Maastricht University, Maastricht, Netherlands Antilles; 2 University of East Anglia School of Health Sciences (Ringgold ID: 14266), Norwich, England, UK

The revised version shows increased clarity and the authors incorporated earlier feedback well. The protocol is ready to get started with the study. I look forward to reading the findings in a later stage.

Is the study design appropriate for the research question?

Partly

Is the rationale for, and objectives of, the study clearly described?

Partly

Are sufficient details of the methods provided to allow replication by others?

Partly

Are the datasets clearly presented in a useable and accessible format?

Yes

Reviewer Expertise:

co-creation of education; SSP; educational psychology

I confirm that I have read this submission and believe that I have an appropriate level of expertise to confirm that it is of an acceptable scientific standard.

# Reviewer report: Reviewer response for version 1

**DOI:** 10.5256/f1000research.179365.r377020

**Published:** 2025-06-27

**Authors:** Karen D Könings

**Affiliations:** 1 Maastricht University, Maastricht, Netherlands Antilles; 2 University of East Anglia School of Health Sciences (Ringgold ID: 14266), Norwich, England, UK

The protocol “Exploring the impact of student-staff partnerships in Higher Education: A Realist Review” describes a literature study proposal aimed at better understanding how student-staff partnership improves the university experiences for all and which challenges are faced and how they are dealt with. The topic of this proposal is interesting and suitable for a realist review study; however, I also see room for improvement, which I will describe in my suggestions for revisions below.

 In the Background section, the authors place considerable emphasis on the local context in Ireland, including an elaborated paragraph about this context upfront in the second paragraph of the protocol. Given that the rest of the protocol is globally relevant, it’s better not to emphasize the context excessively and mention it only in the Methods section.

 Additionally, the Background would benefit from widening the literature review to include knowledge described in papers that use synonyms of SSP to describe similar initiatives (e.g., co-creation, participatory design). In the Background, you neglected this issue, which you did recognize in defining the search terms for the literature search. Better acknowledge it from the beginning. Related to this issue, it appears that you have overlooked relevant literature on challenges and their solutions (Bovill et al., 2016). There is a recent overview of this, from the perspectives of learners, teachers and educational institutions (Könings et al., 2021). It is incorrect to indicate this as a gap in the literature, so this passage needs revision in your text.

 The aim of the study is interesting and important, but in the aim as well as the research question I struggle with the fact that you consider the effects to be applicable for all. In the “Framework of Stakeholder Involvement in Co-Creation” we distinguish between effect for the participating stakeholders in the partnership/co-creation and those who do not directly participate but hopefully benefit from the changes developed in the partnership initiatives. The examples of benefits that you describe are all mainly relevant to the direct participants, as they are related to the improved psychosocial learning environment, and motivation and metacognition. Only improved quality of educational design is likely to affect all. By considering all effects to be equally influencing all, you would overlook the important factor of being direct or indirect affected by SSP.

 The second research question does not logically follow from the introduction, and I wonder what mindsets and modes of engagement are about. Maybe you can keep this more general, i.e. ‘personal characteristics and SSP approaches?

 The argument for conducting a realist review is convincing and clear. There is some repetition in the sections on ‘Realist inquiry’ and ‘Realist review’.

 The proposed method is well described.

 Under ‘Advisory groups’, you stated that preliminary theories will be developed from the “scoping review”. Please clarify which scoping review.

 Table 1 provides a good list of synonyms. What are your arguments to include design-based research as a synonym? Often, these initiatives do not really include learners in the way that SSP aims for.

 In reviewing the studies and extracting the data, have you considered how to distinguish between true partnerships and pseudo-partnerships (Baradell & Bell, 2020)? When conducting a recent scoping review on the impact of SSP on learner well-being, we encountered the challenges of assessing whether learners were genuinely involved in the design and implementation of the initiative. I would recommend developing criteria to follow in the selection process.

 Related to my earlier comment about effects “for all”, in the CMO – Outcomes you’ll have to differentiate between consequences for direct participants and those affected (which can be missing or even contradictory). I hope my suggestions will be helpful in strengthening the protocol. Good luck with the study. I look forward to seeing the results.

1. *Baradell & Bell, 2020 (Ref 1)*
2. *Bovill et al., 2016 (Ref 2)*
3. *Könings et al., 2021 (Ref 3)*

Is the study design appropriate for the research question?

Partly

Is the rationale for, and objectives of, the study clearly described?

Partly

Are sufficient details of the methods provided to allow replication by others?

Partly

Are the datasets clearly presented in a useable and accessible format?

Yes

Reviewer Expertise:

co-creation of education; SSP; educational psychology

I confirm that I have read this submission and believe that I have an appropriate level of expertise to state that I do not consider it to be of an acceptable scientific standard, for reasons outlined above.

## References

[1] : Is health professional education making the most of the idea of ‘students as partners’? Insights from a qualitative research synthesis. *Advances in Health Sciences Education* .2021;26(2) : 10.1007/s10459-020-09998-3 513-580 10.1007/s10459-020-09998-3 33089396

[2] : Addressing potential challenges in co-creating learning and teaching: overcoming resistance, navigating institutional norms and ensuring inclusivity in student–staff partnerships. *Higher Education* .2016;71(2) : 10.1007/s10734-015-9896-4 195-208 10.1007/s10734-015-9896-4

[3] : Learner involvement in the co-creation of teaching and learning: AMEE Guide No. 138. *Medical Teacher* .2021;43(8) : 10.1080/0142159X.2020.1838464 924-936 10.1080/0142159X.2020.1838464 33153367

# Response

**Published:** 2026-01-11

**Authors:** Rachel Farrell

Thank you to **Reviewer 2 **for the detailed and constructive feedback. In response, we revised the manuscript to improve clarity, conceptual precision, and methodological transparency. Specifically, we streamlined and internationalised the **Background** (moving detailed Irish context to the **Methods**) and added broader partnership terminology (e.g., co-creation/co-design/participatory design) to align with our search strategy. We also strengthened the evidence base by incorporating key literature on partnership challenges and responses, and reframed our contribution accordingly. The **aims and research questions** were refined to make the “for whom” dimension explicit, distinguishing outcomes for **direct participants** and **indirect beneficiaries**, and we replaced vague wording in RQ2 with clearer constructs (participant characteristics and SSP approaches). Methodological sections were tightened to reduce repetition, clarify the role of the exploratory scoping phase and advisory group input, delete DBR, and introduce explicit criteria to differentiate authentic partnership from consultative/pseudo-partnership. Finally, we clarified the complementary roles of **PRISMA** and **RAMESES**, and strengthened the search strategy rationale and capture of earlier influential work through citation chasing.

# Reviewer report: Reviewer response for version 1

**DOI:** 10.5256/f1000research.179365.r379618

**Published:** 2025-05-03

**Authors:** Stuart Sims

**Affiliations:** 1 University of Greenwich, London, England, UK

I would like to thank the authors for this submission. The proposal of a Realist Review for Staff Student Partnership should add significant structure and rigour to theory and practice in this area. I particularly welcome the sharing of the protocol through this platform and at this stage to add transparency and the potential for future replicability. As such, I recommend this study protocol is Indexed and look forward to reading the outcomes of the research once it is published. In terms of feedback for the authors, below are some comments and recommendations that I strongly encourage them to consider and revise their protocol as it is currently published accordingly.

 Background:

 The background section generally sets the broad context around SSP well and draws on all of the relevant literature one would expect. It might benefit from some more practical examples of what a SSP interventions or activities might look like.

 I recommend including more information and broader context about Irish HE for an international journal, it only focuses on Ireland in the context of SSP not generally It would be useful to have some evidence to support the claim that many Irish institutions have adopted SSPs E.g. referencing some studies such as ‘Brennan 2012, Reflections on establishing a student–staff partnership in Irish university-based teacher education’, ‘Munro et al 2022 Learning Together in, and about, Student-Staff Partnership in Higher Education’.

 Study objectives and location:

 The aims of the project are worthwhile and relevant, but there were some potential issues with their framing as research questions:

- Q1, there are many variables here which might not overlap, namely that the enablers for students may be different than for staff
- Q1, how is ‘meaningful’ being measured?
- Q1, given the adoption of a broad definition of SSP (which is well rationalised) it might be challenging to place boundaries around ‘what counts’ for inclusion
- Q2, “mindsets” and “modes” are very different things and raise questions of the ability to meaningfully answer this this research question
- Q2, Assumes a uniformity of purpose of SSP, namely ‘improve the university experience for all’ which might not be relevant for all initiatives

Much of the concerns I have about the research questions are fairly well resolved by the methodology, but further clarity of purpose here will help the authors with reporting and for potential readers of the research.

 Methods:

 The rationale for a realist inquiry is well explained and promises an interesting addition to the literature that gets more meaningfully under the skin of partnership.

 CMOC is well explained in general terms but examples linked to HE are somewhat glossed over. As building a rationale for this method is key to distinguish this work amongst a range of other reviews in the field, this could benefit from expansion and identifying more of what this adds to knowledge beyond other review types. A potential area to address is that the examples provided for Realist Reviews seem to focus on more narrowly defined areas, whereas SSP is a principle underpinning many diverse practices.

 When building a rationale for this approach (section: “The use of realist review in higher education”) the authors discuss existing systematic reviews and theoretical pieces but argue a broader study is needed about context. But in doing so they draw upon Realist methodological literature. This feels somewhat circular and tautological in reasoning, namely that a realist methodology justifies itself. Perhaps spending more time unpacking the limitations of the existing SSP reviews might strengthen this. Particularly, as the Mercer-Mapstone et al review for example does address relevant contextual aspects such as positive/negative outcomes and synthesises conclusions around making spaces and engagement practices. The sample search strategy in this protocol seems comprehensive and likely to yield a wealth of relevant publications. The effectiveness of this is a clear distinction between this and prior reviews which is worth emphasising further to defend the original contribution of this protocol.

 I think the protocol would be enhanced with more information about the advisory groups. For the local reference group, further unpacking what types of SSP are undertaken by participants would allow scrutiny of the breadth or specificity of focus. For the Expert Panel, an indication of the members would be helpful too, particularly for F1000 given this journal’s emphasis on transparency in processes and removal of anonymity. But I will leave this to the discretion of the authors.

 General comments:

- CMO 'configuration' and CMO ‘framework’ are both used, I suggest choosing one for consistency
- There are two headings ‘Methods’, please rectify
- For the search strategy, it is worth noting that a number of journals that specialise in SSP (i.e. *International Journal of Students as Partners, Student Engagement in Higher Education, Journal of Education, Innovation, Partnership and Change*) are not associated with traditional publishers and can be inconsistently found on academic databases. I feel this is a limitation of structured reviewing generally rather than this design, but as I am very eager to see the comprehensive results of this review I hope some accommodation is made for this

Is the study design appropriate for the research question?

Partly

Is the rationale for, and objectives of, the study clearly described?

Yes

Are sufficient details of the methods provided to allow replication by others?

Yes

Are the datasets clearly presented in a useable and accessible format?

Not applicable

Reviewer Expertise:

Higher education learning & teaching, staff-student partnership

I confirm that I have read this submission and believe that I have an appropriate level of expertise to confirm that it is of an acceptable scientific standard.

# Response

**Published:** 2026-01-11

**Authors:** Rachel Farrell

Dear reviewer,

 Thank you very much for your valuable and constructive feedback. We have made suggested chages as follows. 

  

- **Added practical examples of SSP activities (e.g., co-design of modules/assessments, student consultant schemes, partnership committees/working groups, co-inquiry) immediately after the partnership definition. **
- **Expanded and internationalised the Irish HE context in Methods, and strengthened claims about SSP uptake using published Irish examples (e.g., Brennan; Munro et al.), while avoiding over-claiming sector-wide prevalence without mapping evidence. **
- **Refined RQ1 to better reflect realist “for whom” logic by distinguishing students and staff and addressing outcomes for direct participants and (where reported) indirect beneficiaries. **
- **Operationalised “meaningful partnership” and linked it to the partnership authenticity criteria applied at full-text screening and recorded during extraction, to strengthen inclusion boundaries. **
- **Reframed RQ2 using clearer constructs (participant characteristics and SSP approaches) and removed implied uniformity of purpose (“improve experience for all”). **
- **Strengthened CMOC explanation by adding a compact higher education–specific CMOC example illustrating how similar SSP practices can produce different outcomes across contexts and stakeholder groups. **
- **Strengthened the rationale for a realist review by explicitly unpacking limitations of existing SSP reviews (including Mercer-Mapstone) and clarifying the distinct contribution of the present review. **
- **Expanded detail on advisory groups, clarifying the types of SSP represented in the local reference group and describing expert panel composition/transparency (subject to consent). **
- **Improved consistency and structure by standardising CMOC terminology and fixing the duplicated Methods heading. **
- **Enhanced the search strategy by adding targeted hand-searching of key SSP journals/websites (to address indexing limitations), alongside forward/backward citation chasing. **
